# Visual rehabilitation in moderate keratoconus: combined corneal wavefront-guided transepithelial photorefractive keratectomy and high-fluence accelerated corneal collagen cross-linking after intracorneal ring segment implantation

**DOI:** 10.1186/s12886-017-0666-1

**Published:** 2017-12-29

**Authors:** Hun Lee, David Sung Yong Kang, Byoung Jin Ha, Jin Young Choi, Eung Kweon Kim, Kyoung Yul Seo, Tae-im Kim

**Affiliations:** 1 Department of Ophthalmology, International St. Mary’s Hospital, Catholic Kwandong University College of Medicine, Incheon, South Korea; 2 The Institute of Vision Research, Department of Ophthalmology, Yonsei University College of Medicine, 50 Yonseiro, Seodaemungu, Seoul, 03722 South Korea; 3 Eyereum Eye Clinic, Seoul, South Korea; 4 Corneal Dystrophy Research Institute, Severance Biomedical Science Institute, Yonsei University College of Medicine, Seoul, South Korea

**Keywords:** Combined corneal wavefront-guided transepithelial photorefractive keratectomy and accelerated corneal collagen cross-linking, Intracorneal ring segment implantation, Keratoconus

## Abstract

**Background:**

To investigate the effects of combined corneal wavefront-guided transepithelial photorefractive keratectomy (tPRK) and accelerated corneal collagen cross-linking (CXL) after intracorneal ring segment (ICRS) implantation in patients with moderate keratoconus.

**Methods:**

Medical records of 23 eyes of 23 patients undergoing combined tPRK and CXL after ICRS implantation were retrospectively analyzed. Uncorrected distance visual acuity (UDVA), corrected distance visual acuity (CDVA), manifest refraction spherical equivalent (MRSE), corneal indices based on Scheimpflug tomography, higher-order aberrations (HOAs), and corneal biomechanical properties were evaluated before and after ICRS implantation, and at 1, 3, and 6 months after combined tPRK and CXL.

**Results:**

There were significant improvements in final logMAR UDVA and logMAR CDVA, and reductions in sphere, MRSE, and all corneal indices from baseline. Significant improvements in logMAR UDVA and reductions in sphere, MRSE, maximal keratometry, keratometry at the apex, mean keratometry, and keratoconus index were noted after ICRS implantation. After tPRK and CXL, significant improvements in logMAR UDVA and logMAR CDVA, and reductions in cylinder and all corneal indices were observed. There were significant improvements in final root mean square HOAs and coma aberrations from baseline, but no changes from baseline after ICRS implantation. Significant reductions in final radius and deformation amplitude from baseline were noted.

**Conclusions:**

Combined tPRK and accelerated CXL after ICRS implantation in moderate keratoconus appears to be a safe and effective treatment, providing an improvement in visual acuity, corneal indices, and HOAs.

**Trial registration:**

retrospectively registered (identification no. NCT03355430). Date registered: 28/11/2017.

## Background

Collagen cross-linking (CXL) is known to alter corneal biomechanics and increase mechanical rigidity by strengthening the corneal tissue, consequently resulting in significant increases in stiffness of the anterior corneal stroma [1]. Patients with keratoconus, ectasia after photorefractive surgery, corneal infections, and chemical burns can benefit from CXL [2–7]. An accelerated CXL protocol, involving application of a higher-intensity light for a shorter period of time, has been developed and is applicable in a variety of clinical settings [8, 9]. Accelerated CXL could halt or slow down the progression of keratoconus, and demonstrates visual and keratometric outcomes comparable to those of conventional CXL [10–13]. Moreover, the shortened treatment time is beneficial for patient comfort and combination of the approach with other therapies including transepithelial photorefractive keratectomy (tPRK), laser in situ keratomileusis (LASIK), or PRK and single intrastromal ring segment implantation for keratoconus treatment [9, 14–16].

Visual rehabilitation has been accomplished through different combinations of intracorneal ring segment (ICRS) implantations, CXL, and/or photorefractive keratectomy (PRK) for keratoconic patients. ICRS implantations act by flattening the central cornea without affecting the corneal visual axis [17, 18]. They have been reported to be effective in reducing mean keratometry values, coma aberrations, and corneal astigmatism [19–21]. Several studies have evaluated the effects of combined CXL and ICRS implantation in patients with keratoconus, and have shown overall additive effects on visual acuity and keratometry values [22, 23]. Combined PRK and CXL have also been used for the treatment of keratoconus [24–27]. A study investigating the effect of topography-guided PRK and CXL after ICRS implantation in patients with low to moderate keratoconus has demonstrated that uncorrected distance visual acuity (UDVA), corrected distance visual acuity (CDVA), keratometry values, and coma aberrations were significantly improved at 6-months postoperatively [28]. Additionally, Coskunseven et al. have reported that, in patients with progressive keratoconus, topography-guided tPRK, after ICRS implantation and followed by CXL, resulted in an improvement in logMAR UDVA, logMAR CDVA, manifest refraction spherical equivalent (MRSE), and mean steep and flat keratometry values [29]. Recently, Zeraid et al. have shown similar results for logMAR UDVA and keratometry values, but demonstrated no significant reduction in coma aberrations after ICRS implantation followed by same-day topography-guided PRK and CXL [30]. Another study has reported that the combination of accelerated CXL and same-day transepithelial phototherapeutic keratectomy and single inferior ICRS is as effective as the combined treatment, using standard CXL, in terms of visual and topographical outcomes [9].

Changes in a variety of corneal biomechanical properties after PRK, LASIK, small incision lenticule extraction, and CXL can be evaluated using the dynamic Scheimpflug analyzer (corneal visualization Scheimpflug technology [Corvis ST], OCULUS, Wetzlar, Germany) [31–34]. This instrument captures the dynamic process of corneal deformation caused by an air puff, using an ultra-high-speed Scheimpflug camera that acquires up to 4330 images per second [34]. Furthermore, recent studies demonstrated that the dynamic Scheimpflug analyzer can be used for differentiating normal eyes from those with keratoconus [35–37].

Because of the positive effects achieved by combinations of these surgical modalities in the treatment of keratoconus, we hypothesized that corneal wavefront-guided tPRK and high-fluence accelerated corneal CXL after ICRS implantation would also show clinical improvement in patients with moderate keratoconus. Additionally, the changes in corneal biomechanical properties during combined corneal wavefront-guided tPRK and corneal CXL after ICRS implantation are not yet fully understood. Therefore, the aim of this study was to evaluate the efficacy, safety, higher-order aberrations (HOAs), and corneal biomechanical properties in patients with moderate keratoconus after ICRS implantation, followed by combined corneal wavefront-guided tPRK and corneal CXL.

## Methods

We performed a retrospective, interventional case series of patients with moderate keratoconus who underwent combined corneal wavefront-guided tPRK and high-fluence accelerated CXL at least 1 month after ICRS implantation from January 2010 to December 2015 at the Eyereum Eye Clinic (Seoul, South Korea). The study adhered to the tenets of the Declaration of Helsinki and followed good clinical practices with the approval of the Institutional Review Board of Yonsei University College of Medicine (Seoul, South Korea). All patients provided informed written consent for their medical information to be included in analysis and for publication. We retrospectively reviewed the medical records of 23 eyes of 23 patients that met the inclusion and exclusion criteria, as defined below.

Combined corneal wavefront-guided tPRK and accelerated CXL after ICRS implantation were performed if a patient was intolerant to contact lenses, had moderate keratoconus without apical scarring, and if progression had been noted over the previous 6 months. All included patients underwent combined corneal wavefront-guided tPRK and CXL at least 1 month (average 2.7 ± 1.1 months; range 1 to 4 months) after ICRS implantation. We excluded patients with central or para-central corneal scarring, central pachymetry <400 μm, corneal endothelial cell density of less than 2000 cells/mm^2^, systemic autoimmune disease, a history of herpetic corneal disease, pregnancy, lactation, or severe dry eye syndrome.

Grading of keratoconus was based on the Amsler−Krumeich classification [38]. Progression was defined as one or more of the following changes over a period of 6 months: an increase of ≥1.00 diopter (D) in maximal keratometry values, an increase of ≥1.00 D in manifest cylinder, and an increase of ≥0.50 D in MRSE.

### Examinations and measurements

Before ICRS implantation (baseline) and after ICRS implantation (before combined tPRK and CXL), and at 1, 3, and 6 months after combined tPRK and CXL, all patients underwent complete ophthalmic examinations, which included examinations for UDVA and CDVA with a Snellen chart (converted to the logMAR scale for statistical analysis), manifest refraction (MR), and autorefraction using the ARK-530A (NCT Nidek Co., Ltd., Aichi, Japan). The safety index was calculated from the final postoperative CDVA/baseline CDVA ratio (in logMAR). The efficacy index was calculated as the final postoperative UDVA/baseline CDVA ratio (in logMAR). Multiple corneal indices were measured at the 8-mm zone using the Scheimpflug tomography system (Pentacam HR; OCULUS).

For measuring changes in corneal aberrations, including HOAs, coma, and spherical aberrations, corneal wavefront analysis was implemented using corneal topographic data obtained with a Keratron Scout topographer (Optikon, Rome, Italy). Root mean square (RMS) values of the corneal HOAs, with analysis up to the 7th order by expanding the set of Zernike polynomials, were calculated.

Corneal biomechanical properties were measured using the dynamic Scheimpflug analyzer at approximately the same time of day. The dynamic Scheimpflug analyzer automatically calculated corneal deformation amplitude, radius values, and maximal concave power when the cornea is deformed to its greatest curvature by the air puff. The deformation amplitude is defined as the maximum amplitude when the cornea is deformed to its greatest concave curvature and is influenced by corneal stiffness [39]. The radius values represent the central concave curvature at the highest concavity (depressed to the highest concavity), while maximal concave power is the inverse radius of the curvature at the highest concavity.

### Surgical technique

As a first step, all patients underwent femtosecond laser-enabled (IntraLase FS; Abbott Medical Optics, Abbott Park, IL, USA) placement of ICRS (Keraring; Mediphacos, Belo Horizonte, Brazil). Segment sizes were determined according to the nomogram provided by the manufacturer. The depth of the ring channels was set at 75−80% of the thinnest pachymetry reading. After surgery, a bandage contact lens (Acuvue Oasys; Johnson & Johnson Vision Care, Inc., Jacksonville, FL, USA) was placed to be removed the next day. Postoperative medication included topical moxifloxacin 0.5% (Vigamox; Alcon Laboratories, Fort Worth, TX, USA) and fluorometholone 0.1% (Santen Pharmaceutical, Osaka, Japan).

After at least 1 month (average 2.7 ± 1.1 months; range 1 to 4 months), all patients were scheduled for combined corneal wavefront-guided tPRK and accelerated CXL treatment. tPRK between the corneal ring segments was performed using an excimer laser (Amaris 1050 Excimer Laser platform; Schwind eye-tech-solutions GmbH and Co KG, Kleinostheim, Germany). The ablation profile was planned using the integrated Optimized Refractive Keratectomy-Custom Ablation Manager software (version 5.1; Schwind eye-tech-solutions GmbH and Co KG). Using this software, ablation was planned based on clinical parameters, including manifest refraction, pachymetry, and corneal wavefront data (up to the 7th order) and topography obtained with the Keratron Scout. The optic zone area of tPRK was 5.8 mm−7.5 mm, and the total ablation zone was up to 8.6 mm.

0.1% riboflavin with hydroxypropyl methylcellulose (Vibex Rapid; Avedro Inc., Waltham, MA, USA) was soaked onto the corneal surface for 10 min immediately after excimer laser ablation. Additional riboflavin solution was added as needed during the soaking process after which was irrigated with 60 cc of chilled balanced saline solution at completion of soaking. UVA exposure (wavelength: 365 nm) was performed with the KXL system (Avedro Inc., USA) which was set to provide a uniform circular diameter of 9.0 mm of irradiation for 360 s at a power of 15 mW/cm^2^ (total dose: 5.4 J/cm^2^) in a 1:1 pulsatile fashion. The cornea was kept wet at 30-s intervals with additional BSS during the irradiation process.

At the end of the surgery, topical levofloxacin 0.5% (Cravit; Santen Pharmaceutical) and fluorometholone 0.1% were administered, a bandage contact lens was placed, and the eye was examined under the slit-lamp. After surgery, topical levofloxacin 0.5% and fluorometholone 0.1% were applied 4 times daily, for 1 month. The dosage was gradually reduced over 3 months.

### Statistical analysis

Results are expressed as mean ± standard deviation. The Shapiro-Wilk test was used to confirm the normality of data. We performed repeated measures one-way analysis of variance (ANOVA) with Bonferroni-adjusted post-hoc comparison to evaluate the differences between parameters in each follow-up period. All statistical analyses were performed using SPSS software version 20.0 (IBM, Armonk, NY, USA). Statistical significance was defined as *P* < .05.

## Results

**Fig. 1:**
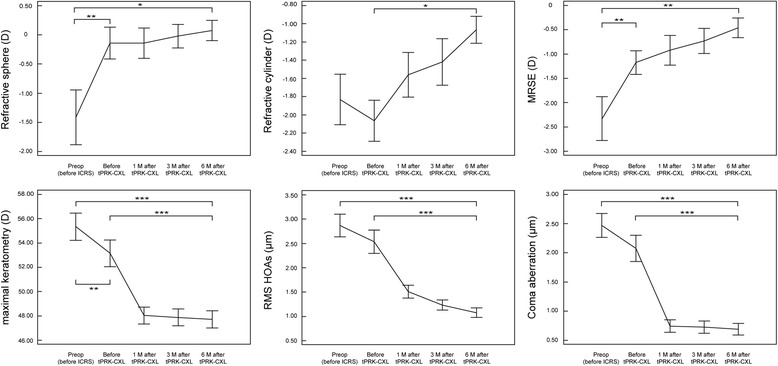
Changes in refractive outcomes, maximal keratometry, and corneal higher-ordrer aberrations in patients with moderate keratoconus who underwent combined corneal wavefront-guided transepithelial photorefractive keratectomy and high-fluence accelerated corneal collagen cross-linking after intracorneal ring segment implantation. Preop = preoperative; ICRS = intracorneal ring segment implantation; tPRK-CXL = corneal wavefront-guided transepithelial photorefractive keratectomy and corneal collagen cross-linking; MRSE = manifest refraction spherical equivalent; RMS HOAs = root mean square higher-order aberrations. Error bars represent standard error of the mean (*P < .05, **P < .01, ***P < .001)

**Fig. 2:**
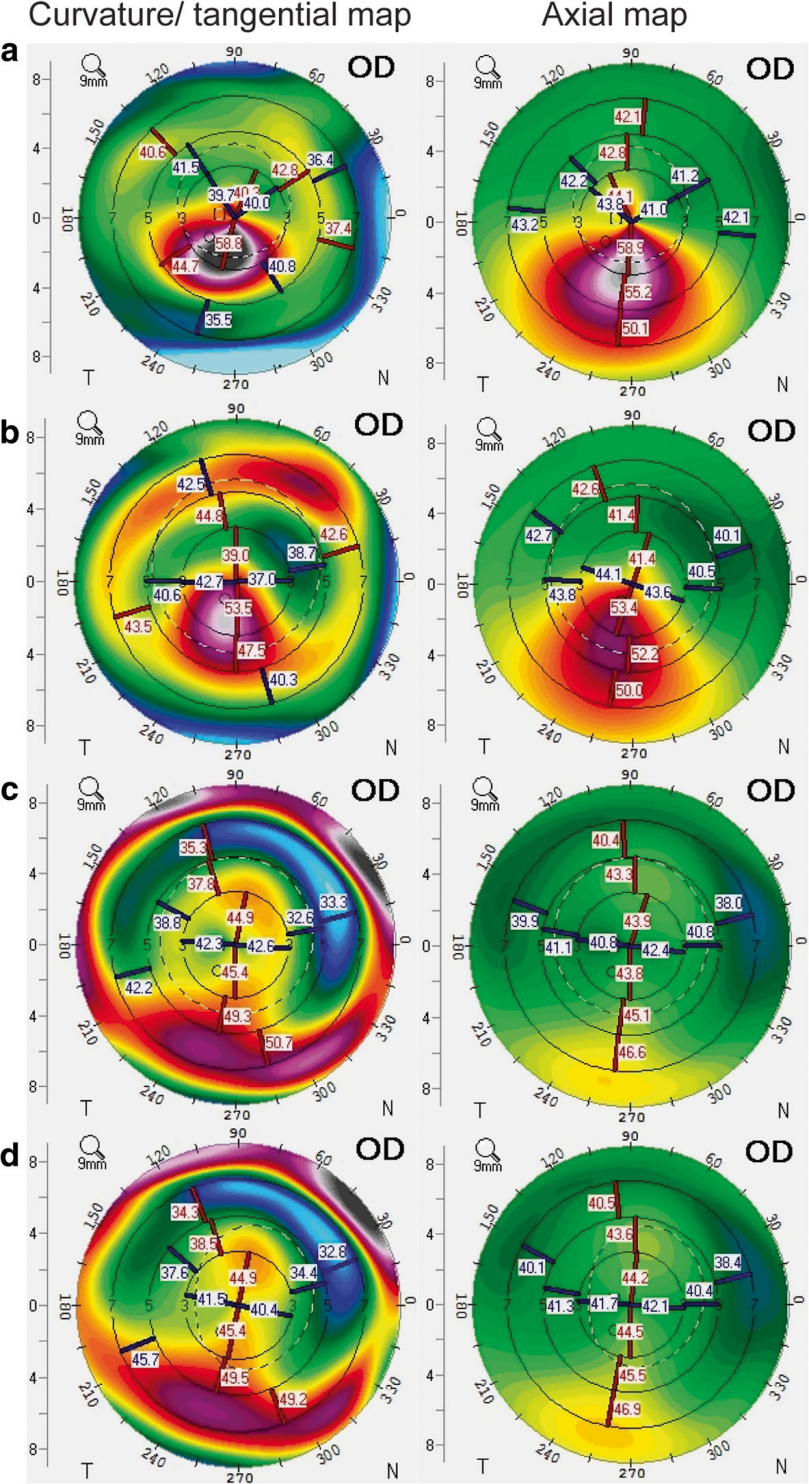
In this patient with moderate keratoconus, combined corneal wavefront-guided transepithelial photorefractive keratectomy (tPRK) and high-fluence accelerated corneal collagen cross-linking (CXL) after intracorneal ring segment (ICRS) implantation achieved a progressive flattening of the cone, as compared to baseline (**a**). Representative corneal topography changes after ICRS implantation (**b**), and at 3 and 6 months after combined tPRK and accelerated CXL (**c** and **d**)

**Fig. 3:**
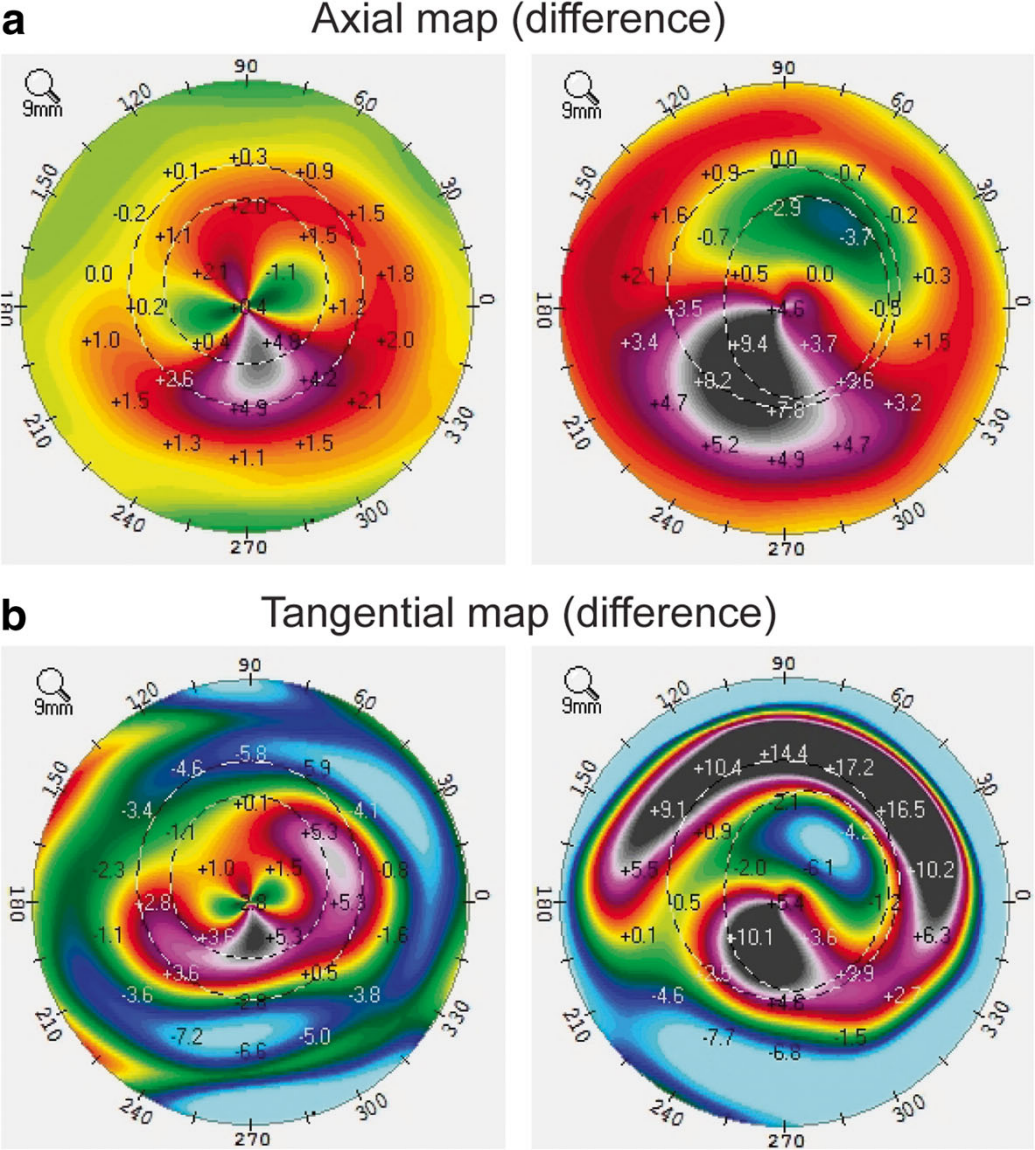
Difference map in patient with moderate keratoconus who underwent combined corneal wavefront-guided transepithelial photorefractive keratectomy (tPRK) and high-fluence accelerated corneal collagen cross-linking (CXL) after intracorneal ring segment (ICRS) implantation. Although the majority of curvature changes occur by combined tPRK and CXL, ICRS implantation also serves to provide 20–30% additive effects. (**a**) axial map (difference), left; after ICRS implantation alone versus before ICRS implantation (baseline), right; 6 months after tPRK and CXL versus after ICRS implantation alone, (**b**) tangential map (difference), left; after ICRS implantation alone versus before ICRS implantation (baseline), right; 6 months after tPRK and CXL versus after ICRS implantation alone

This study included 23 eyes of 23 patients (6 women, 17 men). The mean patient age was 27.1 ± 4.4 years (range: 20−38 years). Table 1 summarizes the baseline patient demographics and clinical characteristics. All surgical procedures were uneventful and no postoperative complications were observed during the observation period. Tables 2 and 3 summarize the postoperative visual acuity, refractive outcomes, and corneal indices before ICRS implantation (baseline), before and at 1, 3, and 6 months after combined tPRK and CXL. After ICRS implantation, there were significant improvements in logMAR UDVA (*P* < .001) and reduction in sphere (MR) (*P* = .002), MRSE (*P* = .002), maximal keratometry values (Kmax) (*P* = .001), keratometry values at the apex (Apex K) (*P* < .001), mean keratometry values (mean K) (*P* = .001), and keratoconus index (KI) (*P* = .002) (Fig. 1). After tPRK and CXL, there were significant improvements in logMAR UDVA (*P* < .001) and logMAR CDVA (*P* = .024), and reduction in cylinder (*P* = .020) and all corneal indices (all *P* < .001) as compared with these values before tPRK and CXL (Fig. 1). There were significant improvements in final logMAR UDVA and logMAR CDVA (all *P* < .001), and reductions in sphere (*P* = .046), MRSE (*P* = .005), and all corneal indices from baseline (all *P* < .001) (Figs. 1, 2 and 3). The safety and efficacy indexes were 0.26 ± 0.27 and 0.89 ± 1.01, respectively. When comparing the differences among 1, 3, and 6 months after combined tPRK and CXL to investigate the effect of different recovery time of visual acuity, logMAR CDVA showed significant improvement between 1 month and 6 months after tPRK-CXL (*P* = .002). The cylinder significantly decreased at 6 months after tPRK-CXL, when compared with 1 month after tPRK-CXL (*P* = .023). After ICRS implantation, there was no significant reduction in any corneal aberrations. However, after tPRK and CXL, there were statistically significant reductions in RMS HOAs and coma aberrations (both *P* < .001) as compared with these values before tPRK and CXL. There were also significant improvements in final RMS HOAs and coma aberration values from baseline (both *P* < .001; Table 4, Fig. 1). When comparing the differences among 1, 3, and 6 months after combined tPRK and CXL to investigate the effect of different recovery time of corneal HOAs, the RMS HOAs significantly decreased during the follow up period (*P* = .025 for 1 month vs 3 months, *P* < .001 for 1 month vs 6 months, and *P* < .001 for 3 month vs 6 months; Table 4). The spherical aberration showed significant difference between 1 month and 6 months after tPRK-CXL (*P* = .008).

**Table 1:** Characteristics of eyes undergoing combined corneal wavefront-guided transepithelial photorefractive keratectomy and accelerated corneal collagen cross-linking after intracorneal ring segment implantation in patients with moderate keratoconus

Characteristics	23 eyes of 23 patients
Age, years old	27.1 ± 4.4 (20 to 38)
Sex (% women)	26%
Refractive errors (D)	
Sphere	−1.41 ± 2.30 (−7.50 to 2.75)
Cylinder	−1.83 ± 1.37 (−5.00 to 0.00)
MRSE	−2.33 ± 2.22 (−8.00 to 1.25)
Keratometric value	
Flat K	46.5 ± 3.2 (41.3 to 56.3)
Steep K	48.0 ± 3.6 (43.3 to 57.8)
Mean K	47.2 ± 3.1 (42.5 to 55.0)
logMAR UDVA	0.85 ± 0.27 (0.30 to 1.30)
logMAR CDVA	0.25 ± 0.18 (0.10 to 0.70)
Optical zone (mm)	6.84 ± 0.36 (6.26 to 7.50)
Total ablation zone (mm)	7.88 ± 0.48 (6.89 to 8.59)
Ablation depth (μM)	34.17 ± 11.60 (14.23 to 54.85)
CCT (μM)	463.9 ± 30.5 (415.0 to 541.0)

Results are expressed as means ± standard deviation (range)

*D* diopters, *MRSE* manifest refraction spherical equivalent, *K* keratometry, *UDVA* uncorrected distance visual acuity, *CDVA* corrected distance visual acuity, *CCT* central corneal thickness

**Table 2:** Preoperative and postoperative visual acuity and refractive outcomes in eyes undergoing combined corneal wavefront-guided transepithelial photorefractive keratectomy and accelerated corneal collagen cross-linking after intracorneal ring segment implantation in patients with moderate keratoconus

	Preop (Baseline, before ICRS)	Before tPRK-CXL	*P* ^a^	1 mon after tPRK-CXL	3 mon after tPRK-CXL	*P* ^b^	6 mon after tPRK-CXL	*P* ^c^	*P* ^d^	*P* ^e^	*P* ^f^
logMAR UDVA	0.85 ± 0.27 (0.30 to 1.30)	0.58 ± 0.27 (0.18 to 1.10)	< .001	0.29 ± 0.29 (−0.20 to 1.20)	0.37 ± 0.30 (−0.10 to 0.80)	.999	0.17 ± 0.14 (−0.10 to 0.54)	.169	.038	<.001	<.001
logMAR CDVA	0.25 ± 0.18 (0.10 to 0.70)	0.19 ± 0.20 (0.00 to 0.70)	.898	0.13 ± 0.07 (0.00 to 0.30)	0.10 ± 0.07 (0.00 to 0.20)	.092	0.07 ± 0.06 (0.00 to 0.20)	.002	.109	<.001	.024
Refractive errors (D)											
Sphere	−1.41 ± 2.30 (−7.50 to 2.75)	−0.14 ± 1.33 (−3.75 to 2.25)	.002	−0.14 ± 1.28 (−4.75 to 1.50)	−0.02 ± 0.99 (−2.75 to 1.25)	.999	0.08 ± 0.85 (−2.00 to 1.25)	.999	.999	.046	.999
Cylinder	−1.83 ± 1.37 (−5.00 to 0.00)	−2.06 ± 1.10 (−5.25 to −0.50)	.999	−1.56 ± 1.21 (−5.00 to −0.25)	−1.42 ± 1.25 (−5.00 to −0.25)	.999	−1.07 ± 0.73 (−3.00 to −0.25)	.023	.145	.059	.020
MRSE	−2.33 ± 2.22 (−8.00 to 1.25)	−1.17 ± 1.19 (−4.50 to 1.00)	.002	−0.92 ± 1.50 (−5.63 to 0.88)	−0.73 ± 1.26 (−4.50 to 0.75)	.999	−0.46 ± 0.99 (−3.50 to 0.75)	.131	.119	.005	.280

Results are expressed as means ± standard deviation (range)

*Preop* preoperative, *ICRS* intracorneal ring segment implantation, *tPRK-CXL* corneal wavefront-guided transepithelial photorefractive keratectomy and corneal collagen cross-linking, *UDVA* uncorrected distance visual acuity, *CDVA* corrected distance visual acuity, *MRSE* manifest refraction spherical equivalent

^a^ *P* value between baseline and before tPRK-CXL

^b^ *P* value between 1 month and 3 months after tPRK-CXL

^c^ *P* value between 1 month and 6 months after tPRK-CXL

^d^ *P* value between 3 months and 6 months after tPRK-CXL

^e^ *P* value between baseline and 6 months after tPRK-CXL

^f^ *P* value between before tPRK-CXL and 6 months after tPRK-CXL

**Table 3:** Preoperative and postoperative corneal indices in eyes undergoing combined corneal wavefront-guided transepithelial photorefractive keratectomy and accelerated corneal collagen cross-linking after intracorneal ring segment implantation in patients with moderate keratoconus

	Preop (Baseline, before ICRS)	Before tPRK-CXL	*P* ^a^	1 mon after tPRK-CXL	3 mon after tPRK-CXL	*P* ^b^	6 mon after tPRK-CXL	*P* ^c^	*P* ^d^	*P* ^e^	*P* ^f^
Kmax (D)	55.35 ± 5.51	53.16 ± 5.39	.001	48.04 ± 3.40	47.87 ± 3.43	.914	47.71 ± 3.47	.143	.389	<.001	<.001
Apex K (D)	55.27 ± 5.30	51.56 ± 6.05	<.001	45.98 ± 4.18	45.53 ± 4.07	.999	45.40 ± 4.00	.999	.999	<.001	<.001
Mean K (D)	46.62 ± 2.87	45.03 ± 2.82	.001	43.16 ± 2.78	42.99 ± 2.83	.999	42.93 ± 2.84	.999	.999	<.001	<.001
ISV	85.48 ± 29.26	76.83 ± 23.09	.365	53.43 ± 17.67	50.78 ± 14.55	.348	50.13 ± 14.28	.167	.999	<.001	<.001
IVA	1.01 ± 0.32	0.87 ± 0.29	.214	0.45 ± 0.26	0.45 ± 0.23	.999	0.44 ± 0.22	.999	.999	<.001	<.001
KI	1.24 ± 0.10	1.18 ± 0.07	.002	1.08 ± 0.07	1.07 ± 0.07	.999	1.05 ± 0.06	.130	.999	<.001	<.001
Pachy (μm)	464.74 ± 31.28	469.96 ± 37.15	.752	412.04 ± 35.33	419.26 ± 31.46	.551	434.61 ± 25.34	<.001	<.001	<.001	<.001

Results are expressed as means ± standard deviation

*Preop* preoperative, *ICRS* intracorneal ring segment implantation, *tPRK-CXL* corneal wavefront-guided transepithelial photorefractive keratectomy and corneal collagen cross-linking, *Kmax* maximal keratometry, Apex *K* keratometry at the apex, *Mean K* mean keratometry, *ISV* index of surface variance, *IVA* index of vertical asymmetry, *KI* keratoconus index

^a^ *P* value between baseline and before tPRK-CXL

^b^ *P* value between 1 month and 3 months after tPRK-CXL

^c^ *P* value between 1 month and 6 months after tPRK-CXL

^d^ *P* value between 3 months and 6 months after tPRK-CXL

^e^ *P* value between baseline and 6 months after tPRK-CXL

^f^ *P* value between before tPRK-CXL and 6 months after tPRK-CXL

**Table 4:** Preoperative and postoperative corneal higher-ordrer aberrations in eyes undergoing combined corneal wavefront-guided transepithelial photorefractive keratectomy and accelerated corneal collagen cross-linking after intracorneal ring segment implantation in patients with moderate keratoconus

	Preop (Baseline, before ICRS)	Before tPRK-CXL	*P* ^a^	1 mon after tPRK-CXL	3 mon after tPRK-CXL	*P* ^b^	6 mon after tPRK-CXL	*P* ^c^	*P* ^d^	*P* ^e^	*P* ^f^
RMS HOAs (μm)	2.87 ± 1.16 (0.28 to 5.97)	2.54 ± 1.18 (0.23 to 5.83)	.999	1.51 ± 0.65 (0.60 to 2.96)	1.23 ± 0.51 (0.52 to 2.39)	.025	1.08 ± 0.49 (0.45 to 2.28)	< .001	< .001	< .001	< .001
Spherical Aberration (μm)	0.15 ± 0.58 (−1.32 to 0.88)	0.03 ± 0.45 (−1.13 to 0.59)	.797	−0.13 ± 0.41 (−1.23 to 0.39)	0.35 ± 0.49 (−0.33 to 2.15)	.092	0.30 ± 0.33 (−0.88 to 0.65)	.008	.999	.999	.128
Coma Aberration (μm)	2.47 ± 1.00 (1.27 to 5.58)	2.07 ± 1.11 (0.11 to 5.33)	.102	0.74 ± 0.54 (0.13 to 2.45)	0.72 ± 0.52 (0.11 to 2.13)	.999	0.69 ± 0.49 (0.11 to 2.09)	.999	.789	< .001	< .001

Results are expressed as means ± standard deviation (range)

*Preop* preoperative, *ICRS* intracorneal ring segment implantation, *tPRK-CXL* corneal wavefront-guided transepithelial photorefractive keratectomy and corneal collagen cross-linking, *RMS HOAs* root mean square higher-order aberrations

^a^ *P* value between baseline and before tPRK-CXL

^b^ *P* value between 1 month and 3 months after tPRK-CXL

^c^ *P* value between 1 month and 6 months after tPRK-CXL

^d^ *P* value between 3 months and 6 months after tPRK-CXL

^e^ *P* value between baseline and 6 months after tPRK-CXL

^f^ *P* value between before tPRK-CXL and 6 months after tPRK-CXL

Preoperative and postoperative corneal biomechanical properties, as measured with the dynamic Scheimpflug analyzer, are shown in Table 5. There were significant reductions in final radius (*P* = .012) and deformation amplitude (*P* = .012), and an increase in final maximal concave power (*P* = .005) from baseline. After tPRK and CXL, there was a statistically significant decrease in deformation amplitude (*P* = .042), as compared with these values before tPRK and CXL, without changes after ICRS implantation from baseline (Table 5).

**Table 5:** Preoperative and postoperative corneal biomechanical properties in eyes undergoing combined corneal wavefront-guided transepithelial photorefractive keratectomy and accelerated corneal collagen cross-linking after intracorneal ring segment implantation in patients with moderate keratoconus

	Preop (Baseline, before ICRS)	Before tPRK-CXL	*P* ^a^	1 mon after tPRK-CXL	3 mon after tPRK-CXL	*P* ^b^	6 mon after tPRK-CXL	*P* ^c^	*P* ^d^	*P* ^e^	*P* ^f^
Radius (mm)	5.33 ± 0.98 (3.56 to 8.05)	4.89 ± 0.71 (3.89 to 6.52)	.042	4.34 ± 0.54 (2.98 to 5.10)	4.51 ± 0.65 (3.00 to 5.60)	.093	4.78 ± 0.67 (3.09 to 5.85)	< .001	.023	.012	.999
DA (mm)	1.09 ± 0.13 (0.89 to 1.43)	1.07 ± 0.11 (0.88 to 1.36)	.938	1.08 ± 0.11 (0.91 to 1.39)	1.03 ± 0.10 (0.91 to 1.35)	.014	1.01 ± 0.11 (0.86 to 1.36)	.008	.844	.012	.042
MCP (1/mm)	0.19 ± 0.03 (0.12 to 0.28)	0.21 ± 0.03 (0.15 to 0.26)	.047	0.23 ± 0.03 (0.20 to 0.34)	0.23 ± 0.04 (0.18 to 0.33)	.371	0.21 ± 0.03 (0.17 to 0.32)	< .001	.034	.005	.999

Results are expressed as means ± standard deviation (range)

*Preop* preoperative, *ICRS* intracorneal ring segment implantation, *tPRK-CXL* corneal wavefront-guided transepithelial photorefractive keratectomy and corneal collagen cross-linking, *DA* deformation amplitude, *MCP* maximal concave power

^a^ *P* value between baseline and before tPRK-CXL

^b^ *P* value between 1 month and 3 months after tPRK-CXL

^c^ *P* value between 1 month and 6 months after tPRK-CXL

^d^ *P* value between 3 months and 6 months after tPRK-CXL

^e^ *P* value between baseline and 6 months after tPRK-CXL

^f^ *P* value between before tPRK-CXL and 6 months after tPRK-CXL

## Discussion

In the present study, we investigated the effects of combined corneal wavefront-guided tPRK and accelerated corneal CXL after ICRS implantation on the visual acuity, refractive outcomes, corneal indices, HOAs, and corneal biomechanical properties in patients with moderate keratoconus. We demonstrated that combined tPRK and CXL after ICRS implantation is beneficial for visual rehabilitation in moderate progressive keratoconus.

As a progressive non-inflammatory ectatic disease, keratoconus involves changes in corneal collagen structure and the intercellular matrix, as well as apoptosis and necrosis of keratocytes [40–42]. CXL stabilizes stromal collagen fibers and hardens the structure of the corneal stroma by inducing formation of additional covalent connections between collagen fibers and other molecules. CXL is also reported to result in topographical flattening of a mean of 2.00 D [43]. The recent introduction of prophylactic CXL application, simultaneously performed with LASIK, aims at strengthening the cornea, particularly in highly myopic eyes with a thin residual stroma [44, 45]. Given the flattening and strengthening effects of concurrent prophylactic CXL, CXL halts the progression of keratoconus and stabilizes the cornea for an extended period of time [3, 4, 7, 9, 12, 46].

There have been multiple reports on the combination of ICRS implantation and prophylactic CXL in patients with keratoconus. For example, Chan et al. demonstrated an additive effect of combination of ICRS implantation and CXL on maximal keratometry values and cylindrical error [47]. Improvement in visual acuity and keratometry values after combination of ICRS implantation and prophylactic CXL has also been reported [23]. Moreover, El-Raggal reported that combining ICRS implantation and CXL in a single, same-day session more effectively reduces keratometry values than consecutive ICRS implantation and CXL, as determined at 6 months, under the assumption that the newly dissected corneal channel created by femtosecond laser may result in more riboflavin pooling and exaggerating the flattening effect of CXL [48]. Combination of PRK, ICRS implantations, and CXL is also known to have an additive effect on visual acuity and keratometry values [9, 28, 29] In our study, we performed combined tPRK and CXL after ICRS implantation in patients with moderate keratoconus. Before employing the combined tPRK and CXL, we performed ICRS implantation, which is known to flatten the conic cornea and shift the decentralized corneal apex more centrally. ICRS implantation is thought to allow implementation of tPRK with minimal tissue ablation. We based on previous reports suggested that high-fluence accelerated prophylactic CXL, performed in combination with tPRK, could not only halt the progression of keratoconus, but also correct refractive errors and reduce HOAs in eyes undergoing ICRS implantation.

In the present study, after ICRS implantation, there were significant improvements in logMAR UDVA and reduction in MRSE, Kmax, Apex K, mean K, and KI from baseline. These results agreed with a study reporting increased UDVA and CDVA and decreased spherical equivalent and mean keratometry values after ICRS implantation, before CXL [48]. We also demonstrated that, after combined tPRK and CXL, there were significant improvements in logMAR UDVA and logMAR CDVA, and reduction in all corneal indices, as compared with before tPRK and CXL. These effects were also shown in an earlier study that demonstrated an additive effect of CXL in terms of an increase in UDVA and decrease in keratometry values [48]. All final parameters, except cylindrical error, were significantly improved from baseline, which agreed with other reports [28, 49]. In terms of safety and efficacy, our results demonstrated better outcomes compared to those obtained in previous studies [28].

In the present study, we demonstrated a significant reduction in final RMS HOAs and coma aberrations as compared with values at baseline and before tPRK and CXL. We reported an improvement in logMAR UDVA attributable to lower order aberration correction by tPRK, and improvement in logMAR CDVA with concomitant decrease in corneal HOAs [50, 51]. The main debilitating visual symptoms experienced by patients with keratoconus are reported to be from the predominant coma aberrations, as well as astigmatism and vertical trefoil [20, 52–54]. A recent study showed that logMAR UDVA and keratometry values improved, whereas coma aberrations did not change, after ICRS implantation followed by same-day topography-guided PRK and CXL [30]. On the other hand, in another study investigating the effect of topography-guided PRK and CXL after ICRS implantation in patients with low to moderate keratoconus, final coma aberrations were significantly decreased when compared with from baseline and after ICRS implantation [28]. This was in accordance with our findings. Moreover, we observed a greater reduction in coma aberrations than those previously reported (1.78 μm versus 0.26 μm) [28]. On average, 72.1% of preoperative coma aberrations were reduced at final follow up (from 2.47 μm to 0.69 μm) with RMS HOAs reduced by 62.3% (2.87 μm to 1.08 μm). This larger reduction may be attributable to the transepithelial ablation profile. A fixed 55-μm tPRK ablation in our combinatory approach may assist the correction of coma aberrations originating mainly in the area of the cone where the epithelium is thinnest [55]. In keratoconic eyes, spherical aberrations have been observed to become more negative as the cone bulges more anteriorly [52]. In our study, there was a trend for spherical aberrations to shift to a less hyperprolate corneal shape (0.15 μm to 0.30 μm), albeit not reaching statistical significance, in accordance with decrease in Kmax. This may be due to the limited amount of ablation depth used in the present study that was insufficient to change corneal shape over a larger area.

In terms of corneal biomechanics, our results showed that final deformation amplitude decreased significantly as compared with that at baseline and before tPRK and CXL. Considering that thinner corneas tend to demonstrate higher deformation amplitudes and that this parameter reflects corneal stiffness, high-fluence accelerated CXL appears to be able to strengthen the cornea in keratoconus [39]. Moreover, the deformation amplitude is a parameter that can be measured with high repeatability and reproducibility when evaluating corneal biomechanics [39, 56]. On the other hand, final radius values significantly decreased as compared with values at baseline. Considering that the radius represents the central concave curvature at the highest concavity, these results contradict changes in deformation amplitude. Thus, the results obtained from the dynamic Scheimpflug analyzer in keratoconic corneas should be interpreted with caution. Moreover, associations between corneal biomechanical properties and corneal thickness or intraocular pressure could affect measurements of corneal biomechanics. Furthermore, more sensitive means of quantifying corneal biomechanics or improvements in computation of relevant parameters are essential when using the dynamic Scheimpflug analyzer in keratoconic eyes.

The present study had several limitations, including its retrospective design. Other possible limitations of this study were the relatively small sample size and the lack of a control group. A prospective, controlled long-term, comparative paired-eye study should be performed to validate the current results.

## Conclusions

A combination of corneal wavefront-guided tPRK and accelerated corneal CXL after ICRS implantation is an effective and safe option for correcting mild refractive errors and improving visual acuity, corneal indices, and HOAs in patients with moderate progressive keratoconus.

## Abbreviations

CDVA: Corrected distance visual acuity
CXL: Collagen cross-linking
HOAs: Higher-order aberrations.
ICRS: Intracorneal ring segment
MRSE: Manifest refraction spherical equivalent
tPRK: transepithelial photorefractive keratectomy
UDVA: Uncorrected distance visual acuity
